# Notes and News

**Published:** 1904-08-13

**Authors:** 


### Notes and News.

During their sojourn at Cowes, the King and Queen visited the Haslar Hospital, and the Queen spent a considerable time at Netley Hospital. The Queen was especially interested in the new nursing quarters at Netley.

From Johannesburg it is reported that the Witwatersrand District is now free from the plague.

The post of secretary to the Great Ormond Street Hospital for Children will not be filled until November. Applications for particulars will be supplied by the assistant secretary.

Through the will of the late Mr. James Lewis Smith, the Cardiff and Newport and County Infirmaries, and the Porthcawl Home of Rest will eventually receive £3,000 each.

Any means by which personal interest in hospital work can be aroused is to be welcomed; and it is above all desirable that the working-classes should have an intelligent understanding of the work within hospital walls. Such an understanding must increase the popularity and gain financial support for the institutions. At Burton, the working-men have not been backward in contributing to the local infirmary, but they have had no more opportunity for becoming acquainted with the arrangements of their hospital in its entirety than has the working-man elsewhere, until quite recently, when one of the managers of one of the collieries organised a visit to it. The wards and offices were systematically inspected, and the arrangements fully explained. The interest aroused, and the knowledge acquired, must have a far-reaching effect in the neighbourhood, and we would recommend those who have the welfare of institutions for the sick at heart, to organise similar visits to their hospital, and thus create for it a band of personal friends and workers.

The late Mr. Samuel Bythesea, of Hove, has bequeathed £1,000 to the Royal Alexandra Hospital for Sick Children, Brighton.

The new Campbell Hospital at Portsoy, Aberdeenshire, has been opened for infectious diseases. Towards this Dr. James Campbell contributed £4,000.

The Canadian Medical Association holds its meeting this year at Vancouver, commencing August 23rd. It has been arranged that Mr. Mayo Robson should deliver the address on Surgery. Numerous excursions and entertainments have been arranged in connection with the meeting.

A most successful meeting of the League of Mercy was held last week at Bayham Abbey, near Tunbridge Wells, by the kind invitation of the Marquis Camden (President of the Tonbridge District), Marchioness Camden, and Mrs. Boscawen (Lady President of the district).

A protest is not unnaturally being made against the large numbers of nursing institutions which are collecting in the region of Nottingham Place and Harley Street. A resident of Marylebone, writing in a local paper, says that it is an open secret that infectious cases are received at some of these houses and that this is one of the dangers attending the presence of these homes in the neighbourhood.

Madame Albani has undertaken to give a concert in aid of the Royal Hants County Hospital on October 7th. The hospital is in debt to the extent of £6,597, and has had to draw upon the endowment to meet the cost of the recent extensive improvements, thus reducing the income by about £400. £15,829 of the total cost (£17,257) has been paid, and the present financial position was attributed by the chairman of the annual meeting, held recently, to the increase in the number of patients received for treatment. Six years ago the numbers were less by one-half than they have been during the past twelve months. The number of patients dealt with in the wards during the last quarter was 360, and there were 1,353 out-patients. The hospital was founded in 1736, and is therefore one of the oldest of the county hospitals.

Within the last week two notable members of the medical profession have passed away. The end of both was sudden, and caused by affections of the heart. Sir William Mitchell Banks, M.D., was born in 1842. He was educated at Edinburgh. He gained various honours and held several posts before becoming F.R.C.S.Eng. He gained a great reputation as a consultant, and was distinguished in cancer research. He served on the General Medical Council and Council of the Royal College of Surgeons. He assisted in the foundation of the Liverpool Royal Infirmary and the Liverpool University and University College. He was knighted in 1899. Dr. T. Gilbert Smith was educated at St. Bartholomew's Hospital and Dublin, where he took his M.D. degree. He was Physician to the London, and the Royal Hospital for Diseases of the Chest. He was a prolific writer and a man of much popularity.

